# Supplementary material for: Access to unauthorized hepatitis C generics: Perception and knowledge of physicians, pharmacists, patients and non-healthcare professionals
Source: PLoS One. 2019 Oct 10;14(10):e0223649. doi: 10.1371/journal.pone.0223649 (PMC6786651; doi:10.1371/journal.pone.0223649)
Supplement: S1 File — Text A.: Fictitious case. A 28-year-old woman diagnosed with hepatitis C who has no access to insurance and does not have the financial resources necessary to personally cover the costs. Table A: Survey. The survey includes 21 questions about the participants’ knowledge and perceptions regarding imported generics and the related risks. Table B: Survey results. Table C: Univariate analysis. (DOCX) [file pone.0223649.s001.docx]

**PLoS One Supporting Information Appendix S1**

Article title: **Access to unauthorized hepatitis C generics: Perception and knowledge of physicians, pharmacists, patients and non-healthcare professionals**

Authors: Amandine Garcia, Sascha Moore Boffi, Angèle Gayet-Ageron, Nathalie Vernaz

The following Supporting Information is available for this article:

**Text A: Fictitious case.** A 28-year-old woman diagnosed with hepatitis C who has no access to insurance and does not have the financial resources necessary to personally cover the costs.

**Table A: Survey.** The survey includes 21 questions about the participants’ knowledge and perceptions regarding imported generics and the related risks.

**Table B: Survey results-**

**Table C: Univariate analysis**

**Text A: Fictitious case**

Good morning, everyone!

As part of my master’s thesis on generic treatments for hepatitis C, I invite you to read the scenario described below and answer the related survey questions, which will require approximately 5 minutes to complete. Your opinion is important and very valuable for this research, and I would like to thank you in advance for your help.

Please note that the scenario described below is fictional. This survey will close on 15 April 2019.

Mrs. C. is a 28-year-old woman who has just arrived in Switzerland without health insurance coverage. She is currently unable to pay the health insurance premiums and does not have access to social assistance.

A few days ago, Mrs. C decided to see a doctor for stomach pain. After having run several tests, the doctor told her that she has chronic viral hepatitis C, a liver disease that can cause cancer and may have other serious consequences. The price of treatment is approximately CHF 30,000, which Mrs. C cannot afford. On the internet, she finds the same treatment priced at CHF 1,000. However, these generic drugs are produced in India and Egypt and they are not authorized for use in Switzerland. Her doctor then tells her about the possibility of importing the generic versions for her personal use through an association recognized as being of public utility that could also help finance these drugs if necessary. A laboratory could verify the quality of these cheaper generic drugs as soon as they arrive in the territory.

What do you think of this scenario?

By completing this survey, you accept that the data collected and analysed anonymously will be used in scientific research. We will not record your email address, and your IP addresses will not be tracked. Thank you very much for your participation!

Amandine Garcia

**Table A: Survey**

| **Respondent characteristics** | |
| --- | --- |
| Gender | □ Female □ Male |
| Categories of age in years | □ 20 - 29 |
|  | □ 30 – 39 |
|  | □ 40 – 54 |
|  | □ >=55 |
| Nationality: | □ Swiss |
|  | □ In European union |
|  | □ Other |
| Education | □ Primary |
|  | □ Postmandatory |
|  | □ University/high level |
|  | □ Other |
| **Importance of efficiency and quality in the choice of treatment** | |
| I know the concept of imported generic drugs | □ Yes |
|  | □ No |
| I think that the original or generic drugs from India and Egypt would be as effective as those produced in Switzerland. | □ I do not agree at all |
|  | □ I do not agree |
|  | □ I agree |
|  | □ I fully agree |
| I think that the original or generic drugs from India or Egypt would all be of the same quality than those produced in Switzerland. | □ I do not agree at all |
|  | □ I do not agree |
|  | □ I agree |
|  | □ I fully agree |
| I don’t care if my original or generic drugs come from other countries as long as they are effective. | □ I do not agree at all |
|  | □ I do not agree |
|  | □ I agree |
|  | □ I fully agree |
| I think that the quality of imported generic medicines must be checked when they arrive in Switzerland. | □ I do not agree at all |
|  | □ I do not agree |
|  | □ I agree |
|  | □ I fully agree |
| **Importance of price in the choice of treatment** | |
| I think that the cost of original or generic drugs has an impact on the choice of treatment. | □ I do not agree at all |
|  | □ I do not agree |
|  | □ I agree |
|  | □ I fully agree |
| I find the CHF 1,000 that the patient will have to pay for imported generic drugs too expensive. | □ I do not agree at all |
|  | □ I do not agree |
|  | □ I agree |
|  | □ I fully agree |
| **Buying medicines on the Internet** | |
| Have you ever bought medicines through the Internet? | □ Yes |
|  | □ No |
| I think medicines purchasing is more accessible via the Internet. | □ I do not agree at all |
|  | □ I do not agree |
|  | □ I agree |
|  | □ I fully agree |
| I think that there’s a risk when buying medicines on the Internet. | □ I do not agree at all |
|  | □ I do not agree |
|  | □ I agree |
|  | □ I fully agree |
| I think that a medicine purchased on a Swiss or foreign website is of lower quality than in Swiss pharmacy. | □ I do not agree at all |
|  | □ I do not agree |
|  | □ I agree |
|  | □ I fully agree |
| I am able to differentiate between a reliable and serious website from a fraudulent website. | □ Yes |
|  | □ No |
| **Buyer’s club and import assistance** | |
| Before this survey, had you ever heard about an association that can help import generics? | □ Yes |
|  | □ No |
| Do you know a buyers’ club? | □ Yes |
|  | □ No |
| If you answered « yes » to the last question, which Buyer’s club do you know? | □ |
| Instead of the patient, I would have liked financial support to import the generic drug from India or Egypt. | □ I do not agree at all |
|  | □ I do not agree |
|  | □ I agree |
|  | □ I fully agree |
| **Additional remarks** | □ |

**Table B: Survey results**

| **I think that the original or generic drugs from India and Egypt would be as effective as those produced in Switzerland** | | | | | | |
| --- | --- | --- | --- | --- | --- | --- |
|  | | ***I agree n/N (%)*** | ***I fully agree n/N (%)*** | ***I do not agree n/N (%)*** | ***I do not agree at all n/N (%)*** | ***Total*** |
| Physicians | | 210/435 (48%) | 43/435 (10%) | 153/435 (35%) | 29/435 (7%) | 435 |
| Patients | | 22/50 (44%) | 3/50 (6%) | 9/50 (18%) | 16/50 (32%) | 50 |
| Pharmacists | | 17/69 (25%) | 5/69 (4%) | 32/69 (46%) | 17/69 (25%) | 69 |
| Non-healthcare professionals | | 115/195 (59%) | 22/195 (11%) | 48/195 (25%) | 10/195 (5%) | 195 |
| **Total** | | **364 (49%)** | **71 (9%)** | **242 (32%)** | **72(10%)** | **749** |
| **I think that the original or generic drugs from India or Egypt would all be of the same quality than those produced in Switzerland.** | | | | | | |
|  | ***I agree n/N (%)*** | | ***I fully agree n/N (%)*** | ***I do not agree n/N (%)*** | ***I do not agree at all n/N (%)*** | ***Total*** |
| Physicians | 133/435 (31%) | | 22/435 (5%) | 238/435 (55%) | 42/435 (10%) | 435 |
| Patients | 18/50 (36%) | | 2/50 (4%) | 13/50 (26%) | 17/50 (34%) | 50 |
| Pharmacists | 9/69 (13%) | | 1/69 (1%) | 38/69 (55%) | 21/69 (30%) | 69 |
| Non-healthcare professionals | 75/195 (38%) | | 11/195 (6%) | 92/195 (47%) | 17/195 (9%) | 195 |
| **Total** | **235 (31%)** | | **36 (5%)** | **381 (51%)** | **97 (13%)** | **749** |
| **I think that the quality of imported generic medicines must be checked when they arrive in Switzerland** | | | | | | |
|  | ***I agree n/N (%)*** | | ***I fully agree n/N (%)*** | ***I do not agree n/N (%)*** | ***I do not agree at all n/N (%)*** | ***Total*** |
| Physicians | 140/437 (32%) | | 289/437 (66%) | 4/437 (1%) | 4/437 (1%) | 437 |
| Patients | 12/50 (24%) | | 36/50 (72%) | 0/50 (0%) | 2/50 (4%) | 50 |
| Pharmacists | 12/69 (17%) | | 53/69 (77%) | 0/69 (0%) | 4/69 (6%) | 69 |
| Non-healthcare professionals | 69/195 (35%) | | 122/195 (63%) | 3/195 (2%) | 1/195 (1%) | 195 |
| **Total** | **233 (31%)** | | **500 (67%)** | **7 (1%)** | **11 (1%)** | **751** |

| **Instead of the patient, I would have liked financial support to import the generic drug from India or Egypt.** | | | | | | | | |
| --- | --- | --- | --- | --- | --- | --- | --- | --- |
|  | ***I agree n/N (%)*** | | ***I fully agree n/N (%)*** | ***I do not agree n/N (%)*** | | ***I do not agree at all n/N (%)*** | | ***Total*** |
| Physicians | 261/430 (61%) | | 93/430 (22%) | 58/430 (13%) | | 18/430 (4%) | | 430 |
| Patients | 26/49 (53%) | | 6/49 (12%) | 7/49 (14%) | | 10/49 (20%) | | 49 |
| Pharmacists | 25/67 (37%) | | 10/67 (15%) | 25/67 (37%) | | 7/67 (10%) | | 67 |
| Non-healthcare professionals | 116/189 (61%) | | 49/189 (26%) | 18/189 (10%) | | 6/189 (3%) | | 189 |
| **Total** | **428 (58%)** | | **158 (21%)** | **108 (15%)** | | **41 (6%)** | | **735** |
|  | | | | | | | | |
| **I am able to differentiate between a reliable and serious website from a fraudulent website** | | | | | | | | |
|  | | ***No*** | | | ***Yes*** | | ***Total*** | |
| Physicians | | 317/431 (74%) | | | 114/431 (26%) | | 431 | |
| Patients | | 29/49 (59%) | | | 20/49 (41%) | | 49 | |
| Pharmacists | | 37/67 (55%) | | | 30/67 (45%) | | 67 | |
| Non-healthcare professionals | | 71/109 (65%) | | | 38/109 (35%) | | 109 | |
| **Total** | | **454 (69%)** | | | **202 (31%)** | | **656** | |

**Table C: Univariate analysis**

|  | **Efficacy** | | **Quality** | | **Quality control** | | **Fraudulent website** | | **Finance initiative** | |
| --- | --- | --- | --- | --- | --- | --- | --- | --- | --- | --- |
| **Variables** | OR  (95%CI) | p-value | OR  (95%CI) | p-value | OR  (95%CI) | p-value | OR  (95%CI) | p-value | OR  (95%CI) | p-value |
| ***Gender*** |  |  |  |  |  |  |  |  |  |  |
| Male | 1 | 0.39 | 1 | 0.602 | 1 | 0.995 | 1 | 0.584 | 1 | 0.392 |
| Female | 1.13 |  | 0.93 |  | 1 |  | 0.91 |  | 1.13 |  |
|  | (0.86-1.48) |  | (0.70-1.23) |  | (0.74-1.36) |  | (0.65-1.27) |  | (0.85-1.51) |  |
| ***Age in years*** |  | **<0.001** |  | 0.06 |  | 0.835 |  | **0.005** |  | 0.055 |
| >=55 | 1 | **-** | 1 | - | 1 | - | 1 | **-** | 1 | - |
| 40-54 | 1.15 | 0.545 | 0.89 | 0.64 | 0.98 | 0.952 | **2.47** | **<0.001** | 1.45 | 0.054 |
|  | (0.73-1.83) |  | (0.55-1.44) |  | (0.58-1.67) |  | **(1.51-4.04)** |  | (0.99-2.12) |  |
| 30-39 | 1.5 | 0.067 | 1.43 | 0.115 | 1.15 | 0.591 | **1.83** | **0.012** | 1.64 | 0.012 |
|  | (0.97-2.31) |  | (0.92-2.23) |  | (0.70-1.89) |  | **(1.14-2.93)** |  | (1.12-2.41) |  |
| 20-29 | **2.31** | **<0.001** | 1.32 | 0.218 | 1.15 | 0.58 | **1.83** | **0.043** | 1.1 | 0.697 |
|  | **(1.48-3.59)** |  | (0.85-2.07) |  | (0.70-1.90) |  | **(1.02-3.28)** |  | (0.67-1.82) |  |
| ***Nationality*** |  | *0.12* |  | *0.575* |  |  |  | *0.266* |  | ***0.004*** |
| Swiss | 1 | - | 1 | - | 1 | 0.745 | 1 | - | 1 | - |
| EU | 0.89 | 0.518 | 1.1 | 0.576 | 0.92 | 0.678 | 1.29 | 0.208 | 1.27 | 0.199 |
|  | (0.63-1.26) |  | (0.78-1.56) |  | (0.63-1.35) |  | (0.87-1.92) |  | (0.88-1.85) |  |
| Other | 0.45 | 0.045 | 0.71 | 0.401 | 0.73 | 0.498 | 1.68 | 0.245 | **0.29** | **0.003** |
|  | (0.21-0.98) |  | (0.32-1.58) |  | (0.29-1.83) |  | (0.70-4.01) |  | **(0.13-0.66)** |  |
| **Education** |  |  |  |  |  |  |  |  |  |  |
| middle level | 1 | 0.18 | 1 | 0.179 | 1 | **0.004** | 1 | 0.663 | 1 | **0.035** |
| high level | 1.57 |  | 1.6 |  | **3.82** |  | 1.22 |  | **2.11** |  |
|  | (0.81-3.03) |  | (0.81-3.20) |  | **(1.52-9.59)** |  | (0.50-2.94) |  | **(1.05-4.24)** |  |
| ***Group*** |  | ***<0.001*** |  | ***<0.001*** |  | ***0.012*** |  | ***0.005*** |  | ***<0.001*** |
| Pharmacists | 1 | **-** | 1 | **-** | 1 | **-** | 1 | **-** | 1 | **-** |
| Physicians | **3.40** | **<0.001** | **3.36** | **<0.001** | **2.40** | **0.005** | **0.44** | **0.002** | **3.16** | **<0.001** |
|  | **(2.10-5.51)** |  | **(2.04-5.53)** |  | **(1.30-4.43)** |  | **(0.26-0.75)** |  | **(1.95-5.11)** |  |
| Patients | **1.67** | **0.165** | 2.03 | 0.066 | 1.54 | 0.322 | 0.85 | 0.671 | 1.64 | 0.178 |
|  | **(0.81-3.45)** |  | (0.95-4.32) |  | (0.66-3.61) |  | (0.40-1.79) |  | (0.80-3.36) |  |
| Non-healthcare | **5.27** | **<0.001** | **4.52** | **<0.001** | **2.75** | **0.002** | 0.66 | 0.191 | **3.47** | **<0.001** |
| professionals | **(3.10-8.94)** |  | **(2.63-7.76)** |  | **(1.44-5.26)** |  | (0.35-1.23) |  | **(2.05-5.87)** |  |
