# Supplementary material for: Access to unauthorized hepatitis C generics: Perception and knowledge of physicians, pharmacists, patients and non-healthcare professionals
Source: PLoS One. 2019 Oct 10;14(10):e0223649. doi: 10.1371/journal.pone.0223649 (PMC6786651; doi:10.1371/journal.pone.0223649)
Supplement: Attachment — Submitted filename: Response to Reviewers 2.docx [file pone.0223649.s002.docx]

**Revised PONE-D-19-21277 entitled “Access to unauthorized hepatitis C generics: Perception and knowledge of physicians, pharmacists, patients and non-healthcare professionals” by Garcia A *et al.***

We would like to thank you and the reviewers for the careful review of our above-mentioned manuscript. We thank the reviewers and the editor for the helpful and constructive comments guiding our revision and the appreciation of the importance of our work.

A point-by-point response to all comments regarding our submission is addressed.

.

**A. Answers to the comments by the editor**

**Editor** 1. To enhance the reproducibility of your results, we recommend that if applicable you deposit your laboratory protocols in protocols.io, where a protocol can be assigned its own identifier (DOI) such that it can be cited independently in the future. For instructions see: <http://journals.plos.org/plosone/s/submission-guidelines#loc-laboratory-protocols>.

We agree to the Plos One recommendation in order to enhance the reproducibility of the results and will therefore provide an excel file of primary data upon acceptance.

**Editor** 2. Please ensure that your manuscript meets PLOS ONE's style requirements, including those for file naming.

We thank the editor for this comment. We now modified our manuscript, title author’s affiliations, main body manuscript including tables, references and supporting information in order to meet PLOS ONE's style requirements.

Former reference 32: “Gustafsson BGBWIBTBJFKGOLJEMCSITCZLL. European payer initiatives to 521 reduce prescribing costs through use of generics. GaBI Journal. 2012;1(1):1:22.” Is now reference 36 and was modified as follow: “Godman B, Wettermark B, Bishop I, Burkhardt; T, Fürst J, Garuoliene K, et al. European payer initiatives to reduce prescribing costs through use of generics. GaBI Journal. 2012;1(1):1:22.”

**Editor** 3. We note that your supplementary file contains a copyrighted image - Dallas Buyers Club image.

We thank the editor for this important comment and have removed the copyrighted image from the supporting information.

**Editor** 4. Please update your ethics statement to include the information on participant anonymity and consent present in your manuscript.

We thank the editor for raising this important issue and added in the marked-up copy the following sentence in line 164, page 9:

“Participation in our survey was voluntary. By completing the survey, all respondents implicitly consented to the use of their data in a scientific study. Because no personal information was collected, no email addresses or IP addresses were logged and all data was collected and analysed anonymously thus guaranteeing the anonymity of all respondents, this study is considered to be exempt from formal institutional review. We added the reference 29: “Article 2 para. 2 of the Federal Act on Research involving Human Beings (HRA) [cited 2019 September 10] Available from: <https://www.admin.ch/opc/en/classified-compilation/20061313/index.html)>.”

**Editor** 5. We note that you have included the phrase “data not shown” in your manuscript. Unfortunately, this does not meet our data sharing requirements. PLOS does not permit references to inaccessible data. We require that authors provide all relevant data within the paper, Supporting Information files, or in an acceptable, public repository.

We thank the editor for this important comment. We have removed the phrase “data not shown” and linked the corresponding results to the corresponding table to the S3 Table: Univariate analysis of the supporting information. The sentence is now as follows in the marked-up copy in line 228, page 14: “In the univariate analysis (see S3Table), we found that the physician and non-healthcare professional groups were consistently associated with all four primary outcomes: compared to pharmacists, physicians and non-healthcare professional were, respectively, 3.40 and 5.27 times more willing to agree regarding the efficacy of imported generics, 3.36 and 4.52 times more willing to agree about their quality, 2.40 and 2.75 times more willing to agree about the need for quality control, and 3.16 and 3.47 times more willing to agree about the need to financially support this initiative.”

**Editor** 6. Please include captions for your Supporting Information files at the end of your manuscript, and update any in-text citations to match accordingly.

This is now addressed in in the marked-up copy line 598, page 33 and updated throughout the manuscript:

Supporting Information

S1 Text A.: Fictitious case. A 28-year-old woman diagnosed with hepatitis C who has no access to insurance and does not have the financial resources necessary to personally cover the costs.

S1 Table: Survey. The survey includes 21 questions about the participants’ knowledge and perceptions regarding imported generics and the related risks.

S2 Table: Survey results-

S3 Table: Univariate analysis

**B. Answers to the comments by the reviewers #1**

Reviewer #1: 1. Please address the current regulations made by the Swiss government to import unauthorized generics and to the Buyer’s Club, and the rationale of allowing a maximum quantity equivalent to one month of therapy at a standard dosage from Swissmedic.

We thank the reviewer or this important comment and modified line 89, page 6 added in the marked-up copy as follows:

“Swiss law allows any individual to import ready-to-use unauthorized medicines for their personal use provided the imported quantity is small and is for their personal consumption.(16, 17) We specified the reference 16 as follow: “Article 20 para. 2 a) of the Federal Act on Medicinal Products and Medical Devices (TPA) [cited 2019 September 10, 2019]. Available from:<https://www.admin.ch/opc/en/classified-compilation/20002716/index.html>.” and added the reference 17: “Article 48 of the Ordinance on licensing of medicinal products (AMBV). [cited 2019 September 10]. Available from: https://www.admin.ch/opc/en/classified-compilation/20180857/index.html.”

We also modified in line 91, page 6 in the marked-up copy as follows:“With the aim of protecting the swiss market and patients from counterfeit or substandard medicines, the regulating authority, the Swiss Agency for therapeutic products or Swissmedic, limits personal imports to a maximum quantity equivalent to one month of therapy at a standard dosage as indicated, for example, in the patient information leaflet.(18)” We added the reference 18: “Swissmedic. Guideline on medicines and the Internet. [cited 2019 February 17]. Available from: https://www.swissmedic.ch/swissmedic/en/home/humanarzneimittel/market-surveillance/medicinal-products-from-the-internet/leitfaden-arzneimittel-aus-dem-internet.html”

Reviewer #1:2. You may cite Liu’s (not ‘Lui’ [44]) another published paper investigating generic DAA for CHC patients on Sci Rep 2018 12;8(1):13699 for reference.

We thank the reviewer for his important comment and modified the phrase in line 325, page 19 “Liu and colleagues”. We also added the following published paper investigating generic DDA for chronic HCV-infected patients “Liu CH, Huang YJ, Yang SS, Chang CH, Yang SS, Sun HY, et al. Generic sofosbuvir-based interferon-free direct acting antiviral agents for patients with chronic hepatitis C virus infection: a real-world multicenter observational study. Sci Rep. 2018;8(1):13699”, reference 37 at line 297 page 18 and line 319 at page 19.

We also added the reference 19: “Freeman JA, Hill A. The use of generic medications for hepatitis C. Liver Int. 2016;36(7):929-32.” In lines 105 and 106 page 9 and line 323 page 19.

Reviewer #1:3. Add ‘analysis’ after ‘univariate’ and delete ‘was’ in the first sentence on page 13.

We thank the reviewer for his comment and modified the phrase as follow in line 228, page 14: “In the univariate analysis (see S3 Table),”

Reviewer #1:4. Polish the language for the 2nd sentence, 2nd paragraph of page 14 (…had the same…than…).

We thank the reviewer for his comment and have sent our manuscript and supporting information to the American Journal Experts for further editing. Line 249, page 15 is now:

“Medical doctors, patients and non-healthcare professionals had all a higher agreement with the statement that Indian/Egyptian generics had the same efficacy as Swiss drugs.”

Reviewer #1:5. The ‘middle level’ educated participants are relatively small in number as compared with ‘high level’ persons, is this true to the general population in Geneva?

We thank the reviewer for this important comment. Due to time constraints and the study horizon, we did not conduct a survey of the general population. We cannot answer this question. This is a limitation and as mentioned an interesting future analysis in line 393, page 22: “to establish to what extent the general population is familiar with these concepts and what measures could be put in place to increase their knowledge and understanding.”

**C. Answers to the comments by the reviewers #2**

We thank the reviewer #2 for his constructive comments and and the appreciation of the importance of our work.

Reviewer #2:1 The details of questionnaire are S1. Did the information of the efficacy and quality of unauthorized generic DAA and real world studies (generic DAA is well-tolerated and provides comparably high SVR12 rates for HCV infection) be introduced before the questionnaire?

We didn’t include any information that might have introduced bias to the survey. The only information the respondents had before completing the survey is addressed in the supporting information (See S1 Text: **Fictitious case**). Please note the same survey was addressed to different stakeholders including non-healthcare professionals and meant to be comprehensive and simple.

Reviewer #2:2. How many the registered physicians at HUF and Lausanne CHUV? The speciality of physicians, such as hepatologist, infectionist ?

The are around 15 hepatologists out of 1370 medical doctors at HUG and around 10 out of around 500 medical doctor who received the survey at CHUV.

3. The manuscript needs linguistic improvement and English editing, such as some redundant sentences, type error.

We thank the reviewer for this comment and have sent our manuscript and supporting information to the American Journal Experts for further editing.

Two redundant sentences were removed:

in lines 79 to 82, page 5: “In this context, prices are reduced significantly in response to intense competition, enabling medicine to be affordable, even for life-saving drugs such as those used to treat the HIV and HCV epidemics”

and in lines 292 and 293, page 17: “However, these drugs represent an important step forward in terms of public health and sustainable financing.“
